# First case report of intestinal lymphangiectasia with refractory bleeding from the duodenum, successfully treated by intra-abdominal lymphaticovenous anastomosis with venous ligation

**DOI:** 10.1007/s12328-024-02021-x

**Published:** 2024-07-17

**Authors:** Yu Miyakawa, Sozaburo Ihara, Saaya Ishii, Yang Rui, Shoh Yajima, Yoku Hayakawa, Yosuke Tsuji, Mutsumi Okazaki, Yasuyuki Seto, Mitsuhiro Fujishiro

**Affiliations:** 1 Department of Gastroenterology, Graduate School of Medicine, The University of Tokyo, Hongo 7-3-1, Bunkyo-Ku, Tokyo, 113-8655 Japan; 2 Department of Plastic and Reconstructive Surgery, Graduate School of Medicine, The University of Tokyo Hongo, Hongo 7-3-1, Bunkyo-Ku, Tokyo, 113-8655 Japan; 3 Department of Gastrointestinal Surgery, Graduate School of Medicine, The University of Tokyo, Hongo 7-3-1, Bunkyo-Ku, Tokyo, 113-8655 Japan

**Keywords:** Intestinal lymphangiectasia, Protein-losing enteropathy, Gastrointestinal bleeding, Lymphaticovenous anastomosis

## Abstract

Intestinal lymphangiectasia (IL) is a protein-losing enteropathy (PLE) that occasionally leads to gastrointestinal bleeding (GIB). We encountered a 41-year-old female with a 9-year history of duodenal IL with PLE and GIB that progressively worsened. Despite a diet, supplemented with medium-chain triglycerides, antiplasmin therapy, oral corticosteroids, octreotides, sirolimus, and repeated endoscopic hemostasis, her symptoms remained uncontrolled, leading to blood transfusion dependence. Lymphangiography revealed significant leakage from abnormal abdominal lymph vessels into the duodenal lumen. The patient subsequently underwent an abdominal-level lymphaticovenous anastomosis combined with local venous ligation. This approach resulted in a dramatic improvement and sustained resolution of both the PLE and GIB. More than 6 months after surgery, the patient remained free of symptoms and blood transfusion dependence.

**Supplementary Information:**

The online version contains supplementary material available at 10.1007/s12328-024-02021-x.

## Introduction

Intestinal lymphangiectasia (IL) is a recognized cause of protein-losing enteropathy (PLE) [1]. Primary IL is often congenital but is also called idiopathic lymphangiectasia because its cause is unknown, whereas secondary intestinal lymphangiectasia is due to lymphatic obstruction [2]. Various factors can lead to lymphatic obstruction and increased lymphatic pressure, including heart surgery, abdominal inflammation, and tumors [3–6]. IL is characterized by the dilatation and leakage of intestinal lymphatics, causing hypoalbuminemia, hypocalcemia, and hypogammaglobulinemia with clinical manifestations of edema, tetany, and recurrent infections [7]. Although treatments, such as dietary therapy, medications, radiological interventions, and surgical procedures, have been reported [8–12], there are no established treatment guidelines for IL. IL is rarely complicated by gastrointestinal bleeding (GIB) [8, 11–13], and some cases do not improve with existing therapies and may be fatal [10]. Here, we present the first case of IL complicated by PLE and refractory chronic GIB that was successfully treated with intra-abdominal lymphaticovenous anastomosis (LVA) with venous ligation in the duodenum.

## Case report

**Fig. 1:**
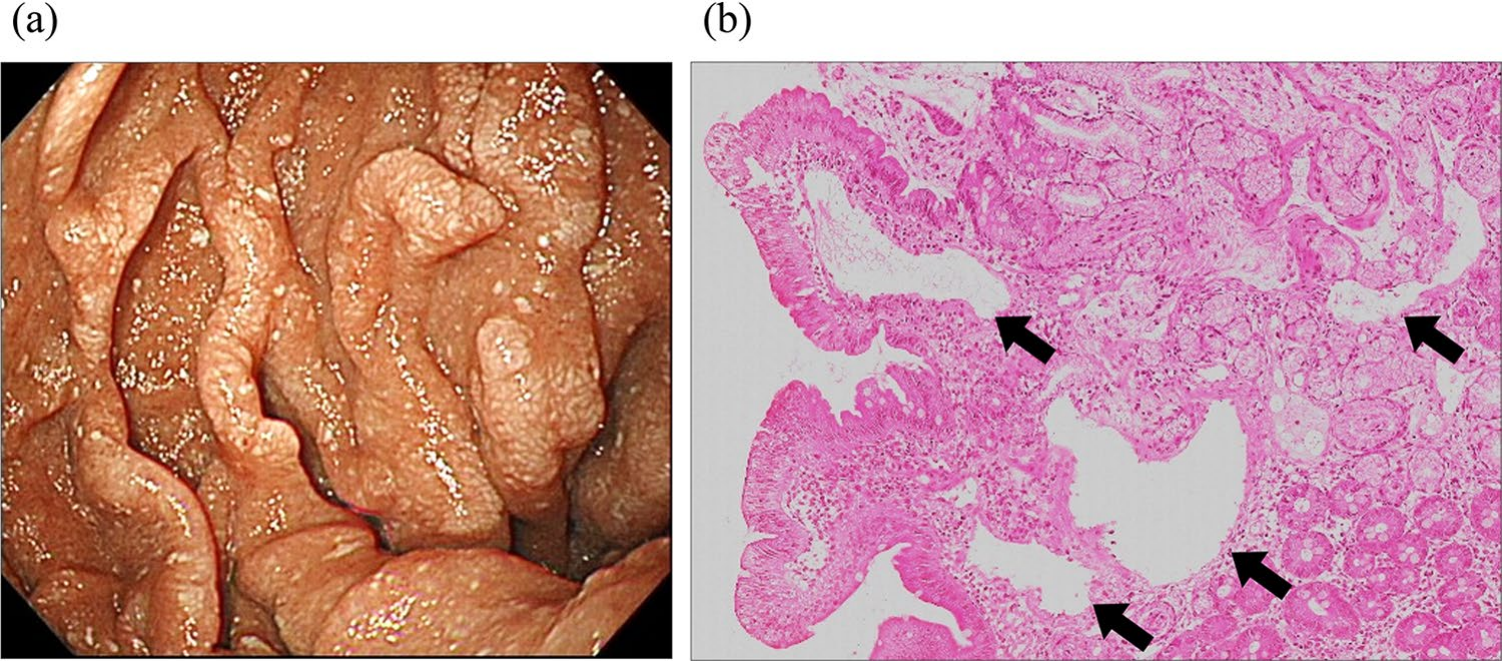
Endoscopic and histological findings at the timing of initial diagnosis. Endoscopy revealed scattered white spots and white villi in the duodenum (**a**), and biopsy of the white villi revealed dilatation of lympahtic vessels in the submucosa. Arrows indicate dilated lymphatic vessels (**b**)

A 41-year-old woman was admitted to our hospital with tarry stool, anemia, and hypoalbuminemia (day 1). She was well until 9 years ago, when she first experienced GIB from minor vascular lesions in the duodenum, which was treated with endoscopic hemostasis. She also developed hypoalbuminemia and was diagnosed with PLE secondary to IL using 99mTc-HSAD scintigraphy (99mTc-human serum albumin-diethylenetriaminepenta-acetic acid). Esophagogastroduodenoscopy (EGD) revealed white spots in the duodenum and dilatation of lymphatic vessels in the biopsies (Fig. 1). She received tranexamic acid for several years, which improved her PLE and GIB, after which she no longer required medical treatment. When she was admitted to our hospital, EGD revealed mild duodenal telangiectasias with a few white spots and minor bleeding, which was controlled using argon plasma coagulation (APC). The patient was subsequently treated with tranexamic acid, diuretics, dietary management, and repeated red blood cell (RBC) transfusions and was discharged. Although she was hospitalized again for pleural and abdominal effusion management with diuretic control (day 45), there was no significant GIB.

**Fig. 2:**
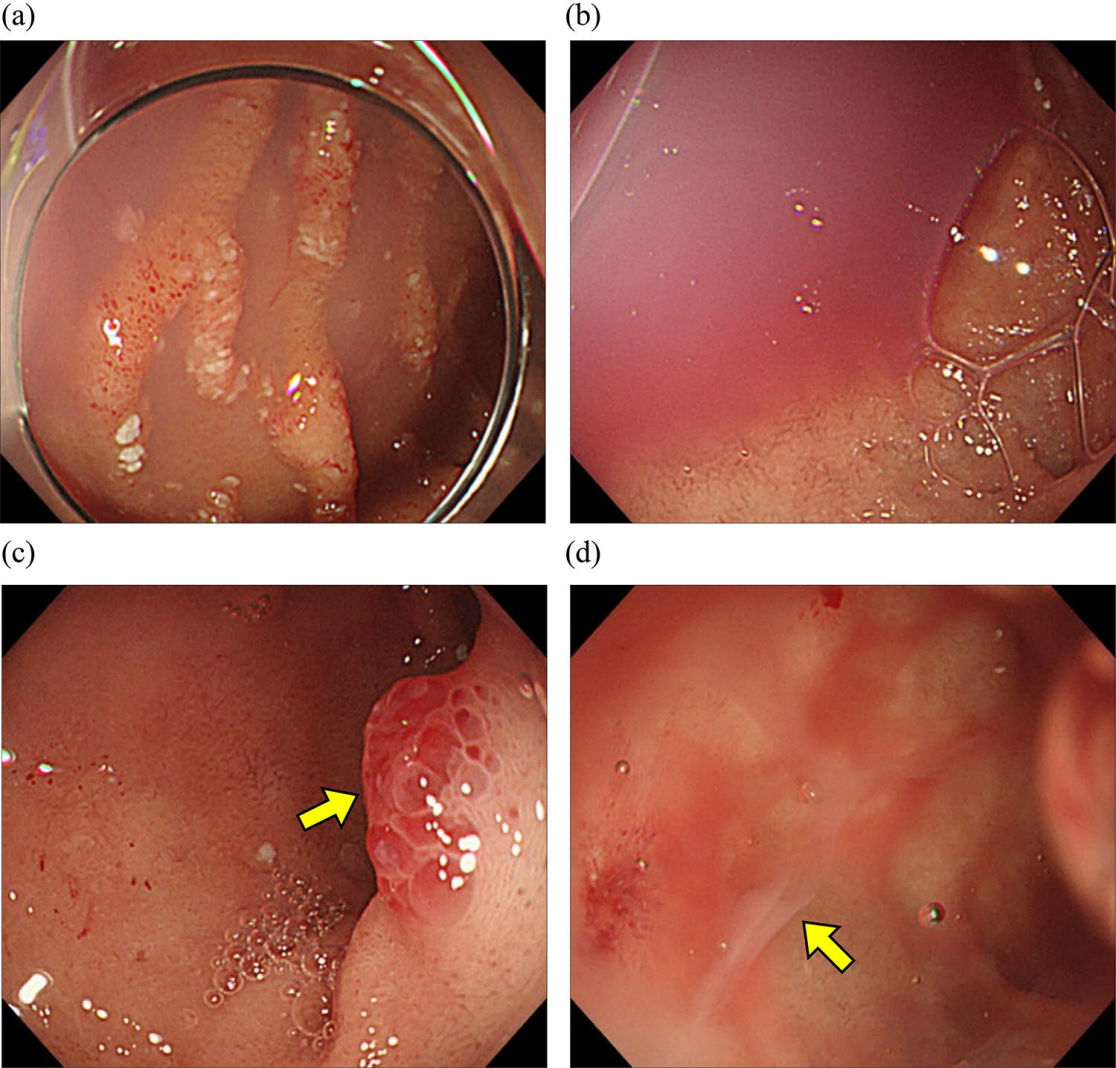
EGD revealed telangiectasia, collection of chylous fluids with pale, scattered white spots in the superior duodenal angle (**a**). The patient did not respond to prednisone treatment, and EGD revealed an exacerbation of a bloody chylous effusion (**b**) with erythematous, granular, and elevated lesions indicated by the arrow (**c**) in the duodenum bulb. Observation by gel immersion showed chylous fluids flowing out (indicated by the arrow) and blood components were also seen nearby (**d**)

Two months later, she was hospitalized for a third time with a chief complaint of tetany in her extremities, and was found to have GIB (day 96). The following laboratory tests were performed: hemoglobin (Hb), 7.2 g/dL; leukocyte count, 4500 with 4.6% lymphocytes; serum calcium, 6.4 mg/dL; serum protein albumin (Alb), 1.7 g/dL, and gamma globulins at 0.22 g/dL. EGD revealed telangiectasias and the collection of chylous fluids with pale blood from the duodenal bulb to the superior duodenal angle, with more pronounced white villi and scattered white spots than at the first time point (Fig. 2a), suggesting an exacerbation of IL with chronic GIB. The tetany improved quickly with Vitamin D and calcium administration; however, chronic GIB was difficult to control, even with multiple APC challenges, and the patient required repeated RBC transfusions.

Although the cause of IL could not be determined at that time, prednisolone 0.6 mg/kg (30 mg), which is known to be effective in cases secondary to inflammation [13, 14], was empirically started on day 109. However, PLE and GIB worsened, and prednisolone was discontinued on day 128. Contrast-enhanced computed tomography, colonoscopy, and capsule endoscopy were also performed; however, no bleeding sites other than the duodenum were detected. EGD revealed increased bloody chylous effusion in the duodenal bulb with an erythematous and elevated granular lesion (Fig. 2b, c). Because bleeding was observed from the anterior duodenal wall on gel immersion gastroscopy (Fig. 2d), APC hemostasis was performed 12 times. However, there was only a minimal response. In contrast, no hemorrhagic findings were observed on EGD of the erythematous elevated lesions on the posterior wall. Because there was no telangiectasia or lymphangiectasia from the intestinal tract distal to the descending duodenal leg, endoscopic procedures including APC for small vessel lesions in the duodenum and hemostatic procedures for erythematous bulging lesions, were performed; however, hemostasis remained insufficient.

**Fig. 3:**
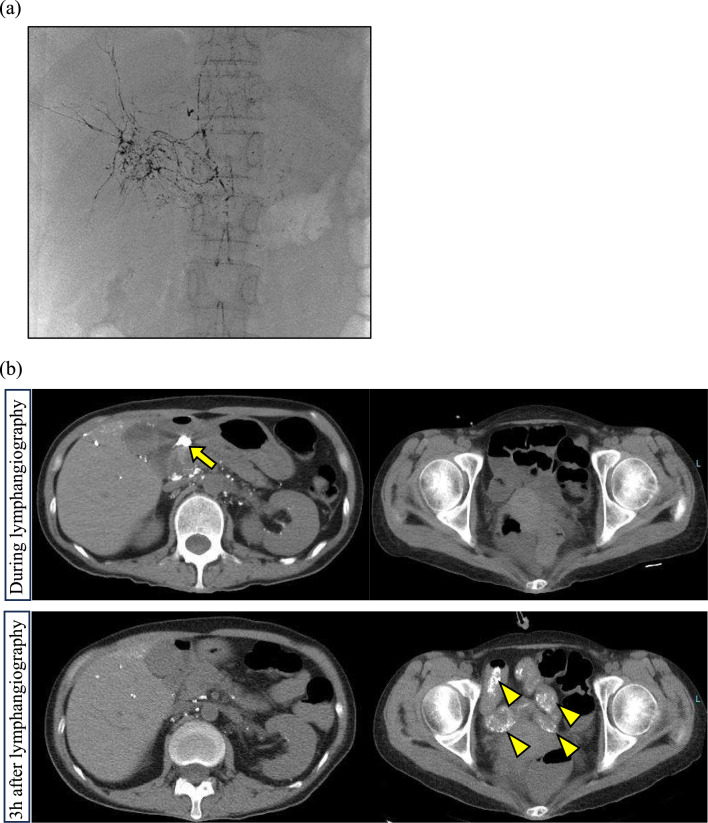
(**a**) Percutaneous transhepatic lymphangiography was performed under ultrasound guidance. A 25G catheter needle was inserted into the Glisson's capsule proximal to the anterior segment of the right lobe of the liver. Contrast medium was then injected, and lymphatic vessels was observed. It showed no obvious cisterna chyle. (**b**) CT images taken during lymphangiography (upper) and three hours later (lower) are shown. During lymphangiography, lymphatic leakage from the posterior wall of the duodenum was observed (arrow). However, after three hours, lipiodol was no longer pooled in the duodenal wall and had flowed into the small intestine (arrowhead)

Considering a possible link between bleeding and lymphatic leakage in the duodenum, octreotide therapy [14, 15] was started on day 156 (initially 200 µg/day, increased to 300 µg/day) but was ineffective in PLE and GIB, and treatment was discontinued on day 177. Following recent reports [2], oral administration of sirolimus (2 µg/day) was started on day 192. Lymphangiography is a valuable tool for detecting lymphatic leakage [16]. Liver lymphangiography is generally used to detect lymphatic leakage originating from hepatic lymphatics such as chylous ascites [17], or suspected lymphatic leakage from the duodenum as seen in some cases of PLE [18]. Therefore, lymphangiography was performed on days 189, 203, and 210 to evaluate lymphatic leakage and the anticipated embolization effects. Imaging conducted through both liver and inguinal lymphangiography showed indistinct lacteals and enlarged periduodenal lymphatic vessels (Fig. 3a). Lipiodol temporarily accumulated at the leak point but was easily expelled into the duodenal lumen, with no embolic effect (Fig. 3b). Images of lymphangiography are shown in Fig. S1 (online resource 1), and CT images taken during lymphangiography are shown in Fig. S2 (online resource 2). The schema of the lymphatic vessels inferred from lymphangiography is shown in Fig. S3. Although blockage of the central lymphatic system was suspected, tests for collagen disease were negative, and cardiac and liver functions were normal. No malignant diseases were detected, suggesting idiopathic lymphatic obstruction.

Despite the expectation of embolization benefits from lymphangiography, we observed significant leakage into the gastrointestinal tract with only minimal lipiodol accumulation around the duodenum, and there was no improvement in PLE and GIB. In this case, because lymphangiography suggested increased intra-abdominal lymphatic pressure and lymphatic flow directed toward the duodenum, an abdominal-level LVA was planned. This procedure focuses on creating a venous anastomosis near the duodenal wall. For GIB, hemostasis was attempted by ligating the target vein in the duodenum. Sirolimus was discontinued on day 244 and a combined LVA creation and venous ligation procedure was performed on day 254.

**Fig. 4:**
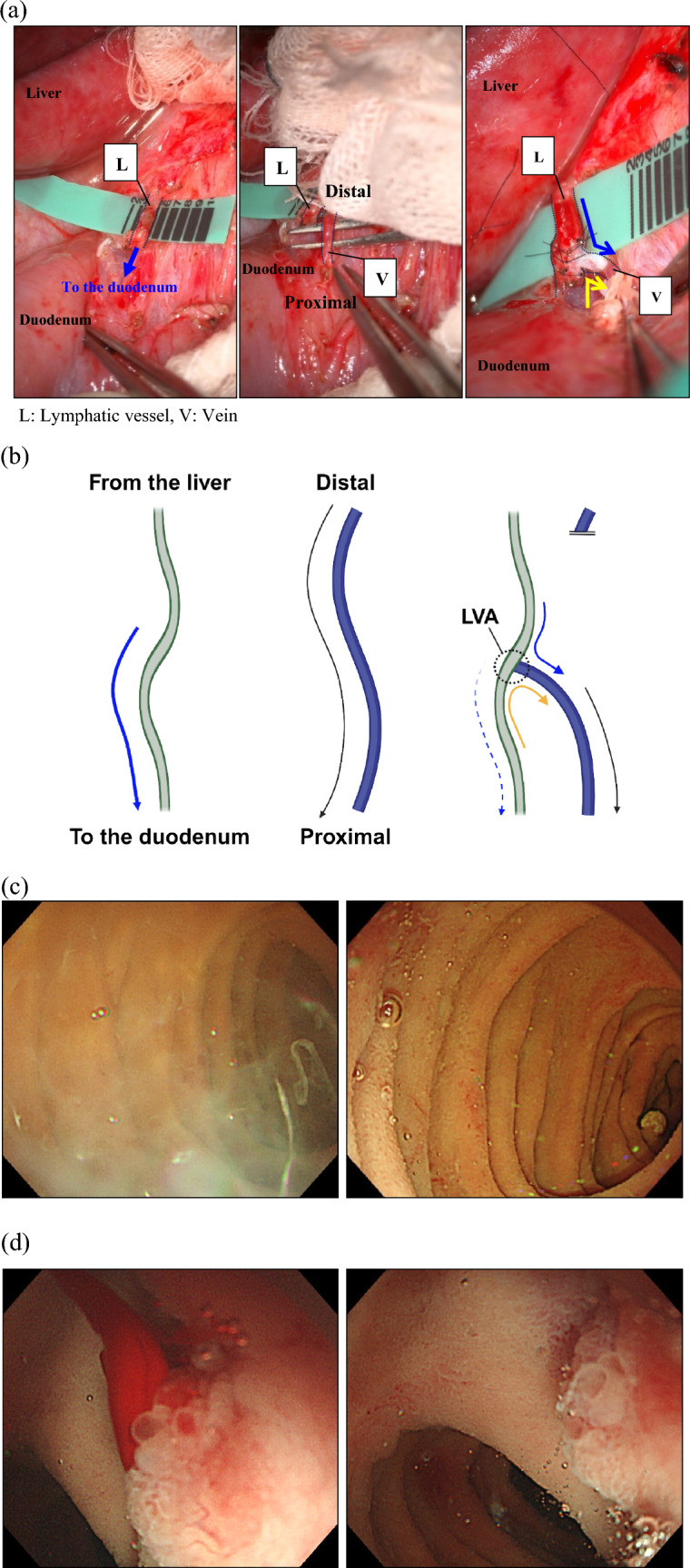
(**a**) A 1.3 mm dilated lymphatic vessel was detected near the proper hepatic artery. The blue arrow indicates the assumed direction of lymphatic flow toward the duodenum (left). A nearby 1 mm vein was detected (center), transected, and the end of the proximal stump was anastomosed to the side of the lymphatic vessel with side-to-end (STE) anastomosis using 9–0 nylon (right). The expected lymphatic flow is indicated by arrows. (**b**) Schema of lymphaticovenous anastomosis by ETS is shown. (**c**) Intraoperative endoscopy showed improvement of duodenal lymphatic leakage before (left) and after (right) LVA. (**d**) Intraoperative endoscopy showed improvement in bleeding from elevated lesion in the posterior duodenal wall before (left) and after (right) duodenal venous ligation

After laparotomy and mobilization of the duodenal bulb, indocyanine green was injected into the serosa of the posterior wall to trace the lymphatic pathways. Intraoperative microscopy revealed that lymphatic vessels originated from the posterior duodenal wall. Subsequently, multiple dilated lymphatic vessels were observed in the ligament area. These are considered the causative lymphatic vessels for LVA. First, a dilated lymphatic vessel on the right side of the proper hepatic artery and a nearby vein were anastomosed to confirm washout (Fig. 4a, b). Next, the lymphatic vessels distal to the duodenum and a nearby vein were anastomosed endo-to-end (ETE) to confirm washout. Finally, a 0.7 mm dilated lymphatic vessel proximal to the duodenum and a nearby vein were anastomosed with the ETE to confirm backflow. During the procedure, several branches of the superior duodenal vein and the infrapyloric vein were thought to contribute to GIB and were ligated. Additionally, lymphatic vessels in the small mesentery were subjected to LVA. However, its dilation was not significant enough to be considered responsible for the excessive lymphatic pressure. Therefore, only one lymphatic vessel in the mesentery and a nearby vein were anastomosed via wrapping. Gel immersion endoscopy, used during the operation for clarity in visualizing the bleeding points [19], helped confirm lymphatic leakage and assess the effectiveness of the LVA (Fig. 4c). Intraoperative endoscopy also allowed the evaluation of bleeding from the duodenum and real-time identification of the bleeding site, followed by additional venous ligation at two sites on the posterior duodenal wall and descending leg (Fig. 4d). Three LVAs were constructed near the posterior duodenal wall and duodenum.

Postoperatively, the patient had a catheter infection, but no other complications. On day 266 (postoperative day 12), the Hb and Alb levels did not decline, the need for a fat-restricted diet was eliminated, and normal food intake was resumed. The patient was discharged on diuretics alone on day 279.

One month post-discharge, the patient's albumin level increased to 3.7 g/dL, the electrolyte imbalance resolved, her Hb improved to 12.7 g/dL, gamma globulin levels rose to 0.6 g/dL, and lymphocyte count reached 400 /µl. Seven months after discharge, her clinical condition remained stable with no signs of lymphatic leakage on EGD, and she no longer required blood transfusions or albumin administration. Trends in Hb and Alb values from initial hospitalization to 7 months after discharge are shown in Table 1.

**Table 1:**
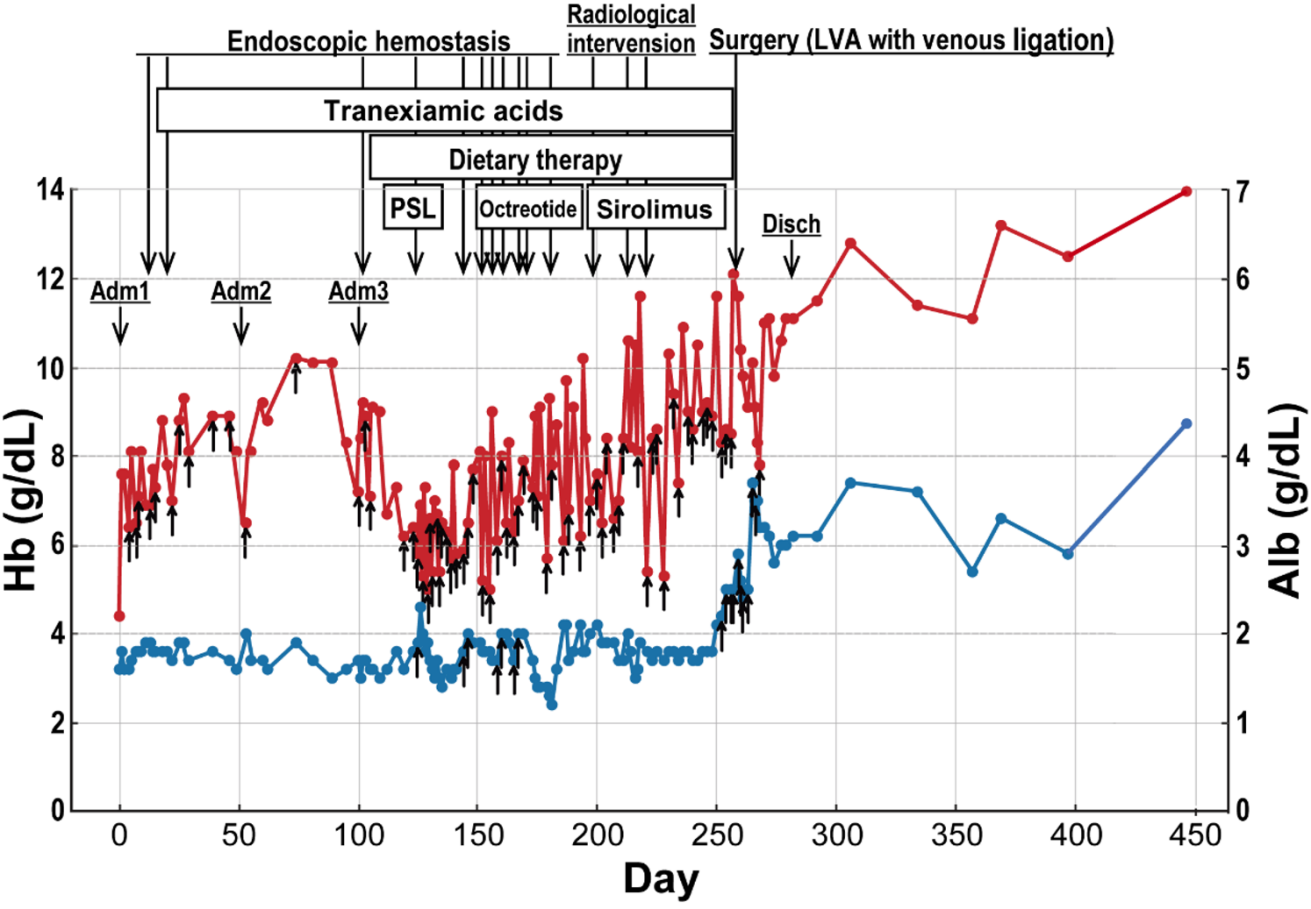
Trends in hemoglobin (red) and serum albumin (blue) levels for the patient. Upper arrows represent the administration of red blood cells (RBC) or serum albumin, respectively. ‘Adm’ indicates admission, and ‘Disch’ indicates discharge. Dietary therapy includes low fat diet, medium-chain triglycerides, and total parenteral nutrition.

## Discussion

Herein, we report a case in which we successfully managed IL complicated by a difficult-to-control GIB. The patient underwent LVA and local venous ligation, resulting in dramatic improvement in hypoalbuminemia due to lymphatic leakage and anemia due to GIB. Cases of IL with GIB have been rare since they were first reported in 1961 [13]. Generally, lymphatic flow in the duodenum drains from the intestinal wall toward the central lacteals. However, when an obstruction at the central level is suspected, as in this case, the lymphatic flow can back up and create local pressure on the intestinal wall [20]. Bleeding may be caused by the rupture of dilated lacteals, which have latent connections with blood vessels [8].

This is the first case report of intraperitoneal LVA with venous ligation of an IL with bleeding. In our case, this treatment approach resulted in improvement without the need for blood transfusions or albumin replacement for up to 4 months after discharge from the hospital. While LVA at the inguinal level has been reported to have a partial effect on severe lower extremity lymphedema in patients with IL [21], this patient did not present with any noticeable leg swelling. Furthermore, given that the primary lesion was located in the central lymphatic vessels, intra-abdominal LVA may be more effective than LVA in the lower leg. During the peeling off of the intra-abdominal vessels, in this case, chylous fluids drained easily from the dilated lymph nodes, necessitating clipping as a preventive measure against lymphatic leakage. Additionally, intraoperative endoscopy to identify bleeding points and efficiently ligate the target vessels has proven to be effective in achieving hemostasis.

In cases where bleeding coexists with local lymphatic leakage, surgical resection of the duodenum or small intestine is a treatment option because hemostasis is expected to be inadequate with endoscopic treatment alone without intervention for lymphatic leakage. Previous reports have documented cases in which surgical resection relieved symptoms without recurrence of bleeding [11, 12]. However, one case has been reported in which surgical resection did not improve the condition and bleeding persisted [10]. Therefore, no treatment guidelines have been established. In this case, neither previously reported medical therapy nor radiological interventions led to improvement; therefore, pancreaticoduodenectomy (PD) was considered first, given the extent of the lesion. However, the opinion of PD was ultimately executed for two reasons. First, a patient's poor clinical condition, including low albumin levels, transfusion dependency, and high risk of infection, increases the likelihood of surgical complications. Second, lymphangiography indicated central lymphatic blockage, suggesting that local surgical resection might lead to the recurrence of lymphatic issues and new gastrointestinal leaks.

In the pediatric field, dietary therapy, followed by octreotide, sirolimus therapy, radiation intervention, and surgery, have been proposed as treatment recommendations for IL [2]. Sirolimus, an inhibitor of the mammalian target of rapamycin (mTOR) signaling pathway, directly affects lymphatic endothelial cells, leading to the inhibition of lymphatic sprouting and proliferation [22]. As in this case, it may be beneficial to continue sirolimus treatment in parallel with radiotherapy and, if the results are unsatisfactory, switch to other treatment modalities, including LVA. In cases where lymphangiography suggests abnormalities in lymph flow or central-level obstruction, constructing an LVA at a more proximal, abdominal level may be effective. Concurrent venous ligation may be a good management strategy, especially when complicated by local GIB, which is difficult to control. This approach may be a treatment option for such cases.

## Supplementary Information

Below is the link to the electronic supplementary material.

Supplementary file1 (MP4 71052 KB)

Supplementary file2 (DOCX 88 KB)

Supplementary file3 (DOCX 241 KB)

Supplementary file4 (DOCX 87 KB)

## Abbreviations

IL: Intestinal lymphangiectasia
PLE: Protein-losing enteropathy
GIB: Gastrointestinal bleeding
LVA: Lymphaticovenous anastomosis
EGD: Esophagogastroduodenoscopy
APC: Argon plasma coagulation
RBC: Red blood cell
