# Supplementary material for: First case report of intestinal lymphangiectasia with refractory bleeding from the duodenum, successfully treated by intra-abdominal lymphaticovenous anastomosis with venous ligation
Source: Clin J Gastroenterol. 2024 Jul 17;17(5):883–90. doi: 10.1007/s12328-024-02021-x (PMC11436469; doi:10.1007/s12328-024-02021-x)
Supplement: Supplementary file 2 — Supplementary file2 (DOCX 88 KB) [file 12328_2024_2021_MOESM2_ESM.docx]

Fig. S3


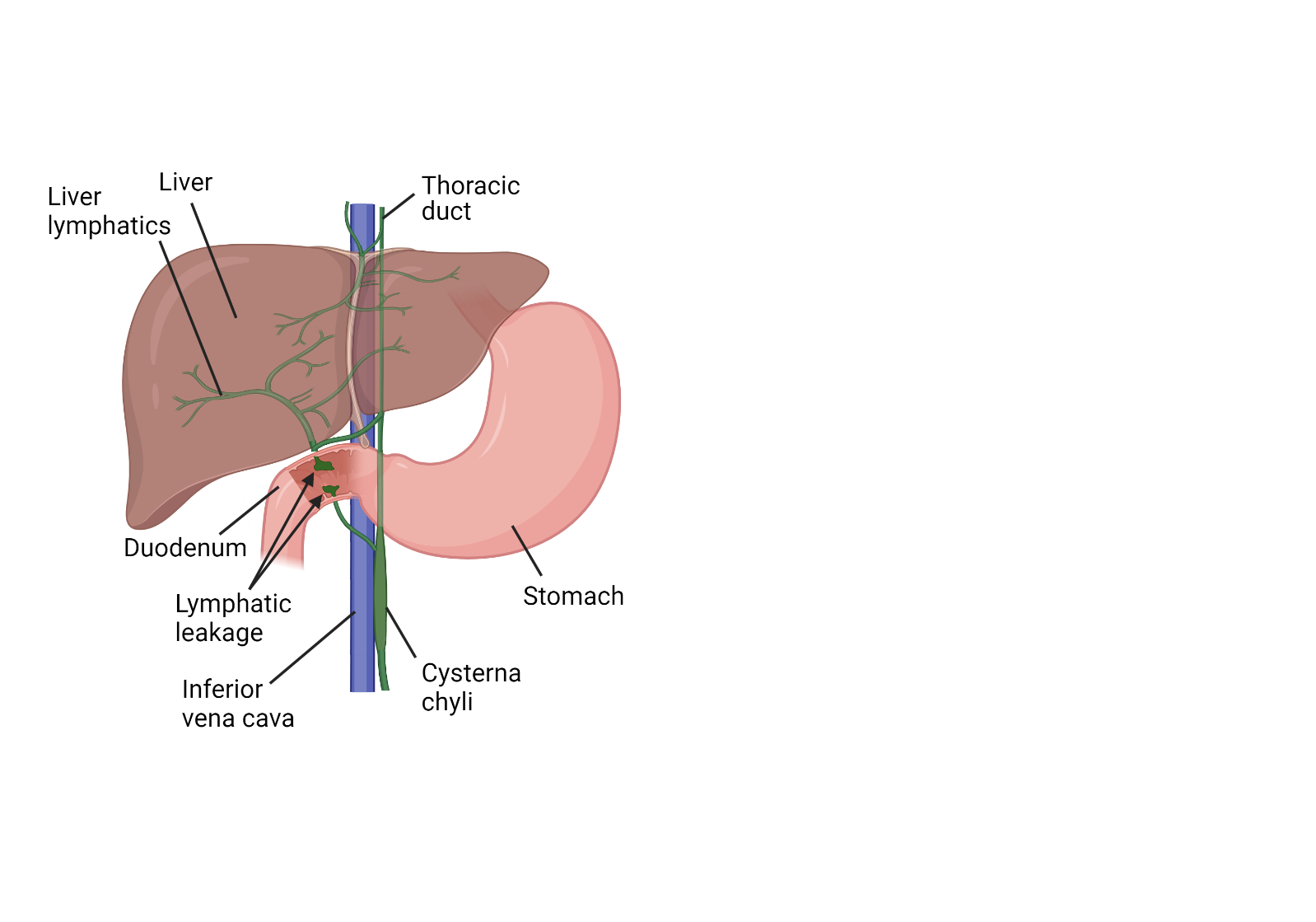


Schema of the lymphatic vessels inferred from lymphangiography. The lymphatic leakage in the duodenal wall was thought to be due to high pressure in the lymphatic vessels, as lymphatic vessels running toward the central side were not clearly seen in this case.
