# Supplementary material for: First case report of intestinal lymphangiectasia with refractory bleeding from the duodenum, successfully treated by intra-abdominal lymphaticovenous anastomosis with venous ligation
Source: Clin J Gastroenterol. 2024 Jul 17;17(5):883–90. doi: 10.1007/s12328-024-02021-x (PMC11436469; doi:10.1007/s12328-024-02021-x)
Supplement: Supplementary file 3 — Supplementary file3 (DOCX 241 KB) [file 12328_2024_2021_MOESM3_ESM.docx]

**Electronic Supplementary Material 2**

Fig. S2 The majority of the main CT images at the liver lymphangiography are shown. Leakage of lipiodol was indicated in the posterior wall of the duodenum, consistent with the findings of the lymphangiogram (Fig. S1).
