# Supplementary material for: First case report of intestinal lymphangiectasia with refractory bleeding from the duodenum, successfully treated by intra-abdominal lymphaticovenous anastomosis with venous ligation
Source: Clin J Gastroenterol. 2024 Jul 17;17(5):883–90. doi: 10.1007/s12328-024-02021-x (PMC11436469; doi:10.1007/s12328-024-02021-x)
Supplement: Supplementary file 4 — Supplementary file4 (DOCX 87 KB) [file 12328_2024_2021_MOESM4_ESM.docx]

**Electronic Supplementary Material 1**

Fig. S1 Lymphangiography showed abdominal lymphatic flow to the duodenum. The lipiodol temporarily stays in the duodenal wall, but leaks into the gastrointestinal tract after a short time.
